# P-669. Comparing Influenza, SARS-CoV-2, and RSV Illnesses among Adults with and without Chronic Medical Conditions

**DOI:** 10.1093/ofid/ofaf695.882

**Published:** 2026-01-11

**Authors:** Shivani Nagapurkar, Oluwakemi Alonge, Emma Viscidi, Joshua Petrie, Jennifer P King, Yoonyoung Park, Gigi Zheng, Catherine A Panozzo, Chelsea Canan, Meng Wang, Wen-Hsing Wu, Iliana Leony Lasso, Jennifer K Meece, Evan J Anderson, Huong Q Nguyen

**Affiliations:** Marshfield Clinic Research Institute, Marshfield, Wisconsin; Marshfield Clinic Research Institute, Marshfield, Wisconsin; Moderna Therapeutics, Cambridge, Massachusetts; Marshfield Clinic Research Institute, Marshfield, Wisconsin; Marshfield Clinic Research Institute, Marshfield, Wisconsin; Moderna, Inc, Belmont, Massachusetts; Moderna, Inc., Cambridge, Massachusetts; Moderna, Inc., Cambridge, Massachusetts; Moderna, Inc., Cambridge, Massachusetts; Moderna, Inc., Cambridge, Massachusetts; Moderna, Inc., Cambridge, Massachusetts; Moderna, Inc., Cambridge, Massachusetts; Marshfield Clinic Research Institute, Marshfield, Wisconsin; Moderna, Inc., Cambridge, Massachusetts; Marshfield Clinic Research Institute, Marshfield, Wisconsin

## Abstract

**Background:**

Older adults and those with chronic health conditions (CHCs) are at increased risk of severe disease from influenza, SARS-CoV-2 and RSV. We assessed illness severity and impact of influenza, RSV and SARS-CoV-2 in adults with and without CHCs.

Overview of Modified Wisconsin Upper Respiratory Symptom Survey – 21 (WURSS-21)

**Figure:**
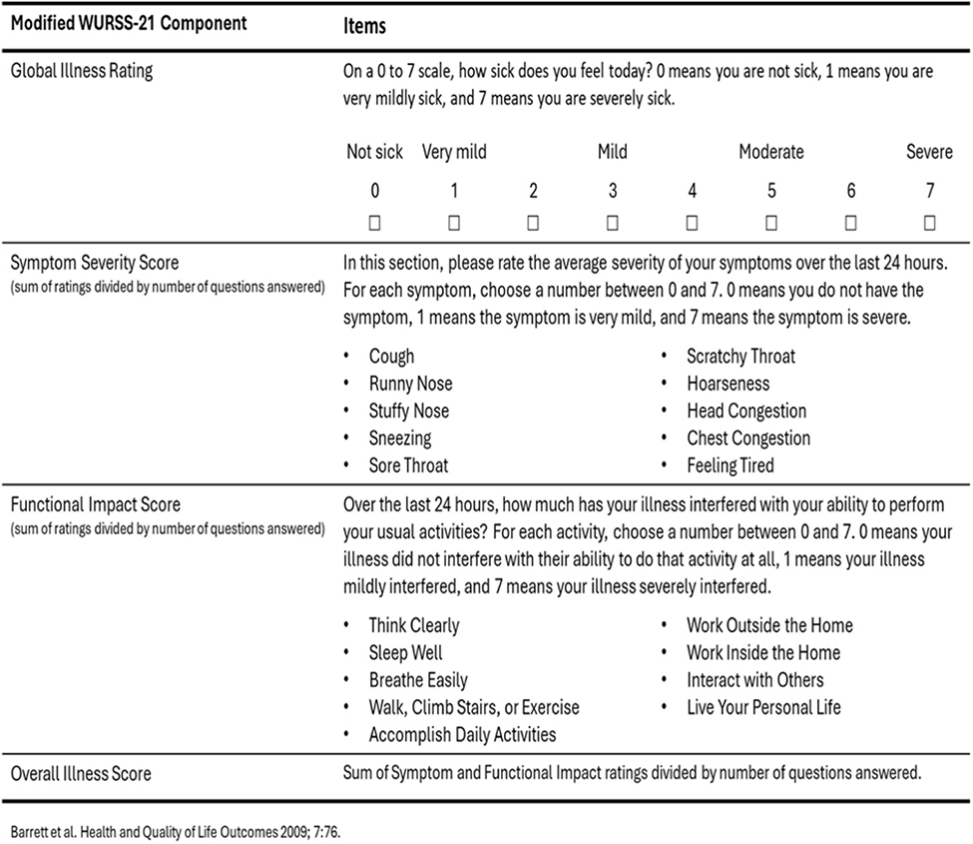

**Methods:**

From September 2024-March 2025, adults seeking inpatient or outpatient care in the Marshfield Clinic Health System for acute respiratory illness were enrolled and tested for influenza, RSV and SARS-CoV-2. A modified Wisconsin Upper Respiratory Symptom Survey (WURSS-21; Table) was used at enrollment to assess overall illness severity, global illness, symptom severity, and functional impact scores. Mean scores were compared by virus using linear regression adjusted for age group, presence of ICD-10-defined CHC, and days from onset to enrollment. Separate unadjusted models compared mean scores for each virus by age/CHC group (18-64 with no CHCs, 18-49 with CHCs, 50-64 with CHCs, and ≥65 with or without CHCs).

**Results:**

Among 1731 adults enrolled, 68% had ≥1 CHC. Influenza, RSV and SARS-CoV-2 were detected in 242 (14%), 67 (4%), and 168 (10%) of adults, respectively; 8 co-detections were excluded. The median (interquartile range) age was 47 (33-65) years for influenza, 58 (46-70) years for RSV, and 55 (36-70) years for SARS-CoV-2 cases. Virus specific vaccination was reported by 70 (33%) influenza cases, 6 (10%) RSV cases, and 34 (24%) SARS-CoV-2 cases. Among 1600 adults enrolled in the outpatient setting, 13% were positive for influenza, 4% for RSV and 10% for SARS-CoV-2. Among 123 hospitalized adults, 24% were positive for influenza, 4% for RSV and 6% for SARS-CoV-2. Mean (95% CI) overall severity scores were higher for influenza (4.0 [3.8-4.2]) and RSV (4.2 [3.8-4.6]) than SARS-CoV-2 (3.7 [3.5-3.9]). Mean (95% CI) symptom severity scores were higher for RSV (4.2 [3.8-4.7]) than both influenza (3.7 [3.5-3.9]) and SARS-CoV-2 (3.6 [3.5-3.9]). Mean global rating (5.0 [4.7-5.2]) vs 4.6 [4.3-4.8]) and functional impact scores (4.5 [4.2-4.8] vs 4.0 [3.7-4.3]) were higher for influenza than SARS-CoV-2. For each virus, mean severity scores were similar by age/CHC groups.

**Conclusion:**

WURSS-21 severity scores differed by virus, but reported illness severity and impact did not differ among adults seeking medical care with and without CHCs for each virus.

**Disclosures:**

Shivani Nagapurkar, MPH, ModernaTX: Grant/Research Support Oluwakemi Alonge, MPH, CPH, CSL Seqirus: Grant/Research Support|GSK: Grant/Research Support|ModernaTX: Grant/Research Support Emma Viscidi, PhD, MHS, ModernaTX: Employee|ModernaTX: Stocks/Bonds (Public Company) Joshua Petrie, PhD, CSL Seqirus: Advisor/Consultant|CSL Seqirus: Grant/Research Support|ModernaTX: Grant/Research Support Jennifer P. King, MPH, GSK: Grant/Research Support|ModernaTX, Inc.: Grant/Research Support Yoonyoung Park, ScD, Moderna: Employee|Moderna: Stocks/Bonds (Public Company) Gigi Zheng, MD, PhD, ModernaTX: Employee|ModernaTX: Stocks/Bonds (Public Company) Catherine A. Panozzo, PhD, ModernaTX: Employee|ModernaTX: Stocks/Bonds (Public Company) Chelsea Canan, PhD, ModernaTX: Employee|ModernaTX: Stocks/Bonds (Public Company) Meng Wang, PhD, ModernaTX: Employee|ModernaTX: Stocks/Bonds (Public Company) Wen-Hsing Wu, MS, Moderna: Stocks/Bonds (Public Company) Iliana Leony Lasso, n/a, ModernaTX: Employee|ModernaTX: Stocks/Bonds (Public Company) Jennifer K. Meece, PhD, CSL Seqirus: Grant/Research Support|GSK: Grant/Research Support|ModernaTX: Grant/Research Support Evan J. Anderson, MD, Moderna: Stocks/Bonds (Public Company) Huong Q. Nguyen, PhD, MPH, CSL Seqirus: Advisor/Consultant|CSL Seqirus: Grant/Research Support|GSK: Grant/Research Support|ModernaTX: Advisor/Consultant|ModernaTX: Grant/Research Support

